# Patterns and predictors of exclusive breastfeeding in Chinese Australian mothers: a cross sectional study

**DOI:** 10.1186/s13006-020-00304-w

**Published:** 2020-07-13

**Authors:** Konsita Kuswara, Karen J. Campbell, Kylie D. Hesketh, Miaobing Zheng, Rachel Laws

**Affiliations:** 1 Institute for Physical Activity and Nutrition, School of Exercise and Nutrition Sciences, Deakin University, Geelong, VIC Australia; 2 Centre for Research Excellence in the Early Prevention of Obesity in Childhood, Charles Perkin Centre, University of Sydney, Sydney, NSW Australia

**Keywords:** Exclusive breastfeeding, Infant formula, Childhood obesity, Chinese immigrant, Factors, Prevalence, Self-efficacy

## Abstract

**Background:**

While exclusive breastfeeding is recommended to 6 months of age, just 15% of mothers in Australia achieve this. The rate appears to be even lower among mothers born in China, where 90% have introduced infant formula by this time. This study aimed to examine infant feeding patterns in the first 12 months of life and the factors associated with exclusive breastfeeding at 4 months of age and infant formula introduction by 1 month of age among Chinese Australian mothers.

**Methods:**

Chinese Australian women with a child aged 1 to 4 years born in Australia were recruited through social media and asked to complete an online survey. Chinese ethnicity was defined as the mother or her parents having been born in mainland China, Taiwan or Hong Kong. Infant feeding practices since birth and a range of psychosocial and cultural factors were assessed. A total of 289 Chinese Australian mothers completed the survey. The relationships between exposure variables and exclusive breastfeeding at 4 months or infant formula use by 1 month were examined using multivariable logistic regression.

**Results:**

Almost all (93%) mothers initiated breastfeeding, however by 1 month of age exclusive breastfeeding rates reduced to 44%, with a further decline to 33 and 18% at 4 and 6 months respectively. Concurrently, 7% of parents reported infant formula feeding at birth increasing to 55 and 63% at 1 and 6 months of age respectively. The rates of any breastfeeding were 81% at 6 months and 50% at 12 months of age. Breastfeeding intention, self-efficacy and awareness of the infant feeding guidelines were key factors associated with sustained exclusive breastfeeding to 4 months.

**Conclusions:**

While Chinese Australian mothers had comparable exclusive breastfeeding rates at 6 months to the general Australian population, twice as many had introduced infant formula by 1 month of age. There is an urgent need to support Chinese Australian mothers in the perinatal period to strengthen their knowledge, intention and confidence to delay early introduction of infant formula and promote exclusive breastfeeding in the early postpartum period.

## Background

The Australian Infant Feeding Guidelines, consistent with the World Health Organization, recommend infants be exclusively breastfed for around 6 months when solid foods are introduced. These guidelines suggest breastfeeding be continued to 12 months or beyond [1, 2]. Exclusive breastfeeding (EBF) is defined as the infant receiving only breast milk, including expressed milk, and vitamin drops or medicine [3]. Adherence to the infant feeding recommendations not only promotes healthy growth and development in infancy but also reduces the risks of overweight and obesity in early childhood and type 2 diabetes in later life [4–6]. In high income countries, including Australia, where metabolic diseases pose a significant burden, improving breastfeeding rates is essential to prevent the early development of excess adiposity and its associated metabolic diseases.

The Australian National Infant Feeding Survey in 2010 showed that the breastfeeding initiation rate in Australia is high at 96%, however at 6 months of age EBF rate reduced to 15%, and at 12 months only 18% of mothers offer any breastfeeding [7]. A secondary analysis of the survey showed that a greater proportion of Chinese born mothers have used infant formula (90% vs. 81%), and on average introduced it at an earlier age than Australian born mothers (1.2 compared to 1.7 months) [8]. Consistent with this, in a secondary analysis of data from a clinical trial at two hospitals in Sydney, Dahlen and Homer [9] reported that Asian born mothers (including Chinese born) were more likely to be partially breastfeeding in the first 12 weeks than non-Asian mothers in Australia. Interestingly, both studies reported that Chinese born (and other Asian ethnicities) mothers were more likely to be breastfeeding across the first year of life than Australian born mothers [8, 9]. Chinese immigrants are the largest culturally and linguistically diverse ethnic group in Australia including 5.6% reported as having a Chinese heritage and 2.6% who were born in China [10]. The term ‘Chinese Australians’ is used in this study to refer to Chinese immigrants from China (including Hong Kong and Taiwan) as distinguished from Chinese from other Asian countries, who are living in Australia. The wide adoption of infant formula, referred to as ‘formula’ from hereon, at an early age in this population group is not consistent with the infant feeding guidelines and warrants further investigation.

A consistent body of evidence in high income countries, including Australia, suggests that optimal breastfeeding practice is associated with breastfeeding intention, self-efficacy and social and professional support [11, 12], while concerns over milk supply are the most frequently reported reason for early cessation of breastfeeding and for introducing formula [13]. Recently, the role of the older generation in influencing breastfeeding outcomes is increasingly recognised. Two systematic reviews including studies from high and low middle income countries showed that grandmothers have an important influence on the parents’ infant feeding decisions [14, 15]. Having a grandmother who had breastfed or had a positive view towards breastfeeding increased the likelihood of the mother breastfeeding exclusively by 1.6 to 12.4 times [14] and strengthened their breastfeeding intention, initiation and duration [15].

Studies among Chinese mothers (native and immigrant) suggest that in addition to psychosocial factors, cultural and familial factors may be significant in shaping Chinese mothers’ breastfeeding beliefs and practices [16]. In the Chinese culture, there is a belief that a woman’s body is weak in the immediate postpartum period resulting in reduced ability to produce sufficient breast milk [17, 18]. The mother is recommended to follow a prescribed ritual of dietary and rest practices to improve recovery and breast milk production. A perception of insufficient milk supply is common and persistent across the first year even in the absence of breastfeeding problems [19]. The emphasis on the woman’s physiological weakness may exacerbate mothers’ anxiety of insufficient milk supply and spur them to supplement with formula. Interestingly, studies with Chinese mothers in Australia have shown that following the Chinese traditional postpartum diet facilitated a longer breastfeeding duration [20, 21].

The majority of the evidence thus far is qualitative, and none of the quantitative studies have examined the relationships between psychosocial, cultural and familial factors with breastfeeding exclusivity or formula introduction. Given the widespread early introduction of formula, it is important to identify modifiable factors to improve EBF rates among Chinese Australian mothers. This study aims to investigate the key psychosocial and cultural factors of early introduction of formula (by 1 month of age) and EBF at 4 months among Chinese Australian mothers. This understanding will inform future culturally appropriate interventions to support Chinese Australian mothers to achieve best infant feeding practices.

## Methods

### Study design and participants

This study utilised a cross sectional survey design and collected data through an online survey between February and August 2018. The survey was developed using Qualtrics software (Provo, UT) to measure infant feeding practices, psychosocial, cultural, demographic, maternal health, child and birth factors. Maternal recall has been shown to be a valid and reliable method to estimate breastfeeding initiation and duration, especially within 3 years of breastfeeding [22]. The online survey was translated into Chinese by an accredited translator and checked independently by a second accredited translator to ensure accuracy. A member of the research team who is a native Chinese speaker further checked the translated survey and no discrepancies were raised. The simplified Chinese survey was also converted into traditional Chinese using an online program (Google Translate™) by replacing simplified Chinese characters with traditional Chinese characters.

Eligible participants were Chinese mothers residing in Australia, aged 18 years and above, with a singleton aged 12–48 months born healthy and full term in Australia. Chinese ethnicity was defined as the mother or her parents having been born in mainland China, Taiwan or Hong Kong. Participants were recruited through online advertisements on social media including paid weekly advertising on Facebook™, guest-posts on the WeChat™ accounts of several Chinese community organisations, and several popular parenting forums for Chinese Australians. Traditional advertising used included distribution of printed flyers in relevant early childhood settings and community venues highly frequented by Chinese people in Melbourne, Australia. Participants could choose to complete the survey in English or Chinese (simplified and traditional) and all provided consent to participate. When participants had multiple children, they were asked to respond to the survey in relation to their youngest child who was aged 12–48 months.

This study was approved by the Human Ethics Advisory Group, Faculty of Health, at Deakin University, HEAG-H 183_2017.

### Measures

#### Outcome measures

##### Infant feeding practices

The primary outcomes of interest were EBF for 6 months and formula introduction in the first month of age. However, due to an insufficient number of mothers exclusively breastfeeding at 6 months (53 participants [18%]), a duration of 4 months was used. Participants were asked how they fed their infant at birth with the options of plain water, sugar or starchy water, colostrum, infant formula, cow’s milk, soy milk or other. Participants who selected options other than colostrum were asked if they had ever breastfed. Other questions included if and at what age they introduced formula, fluids other than breast milk or formula, referred to as ‘other fluids’ from hereon, and solid foods, and when breastfeeding ceased. If participants were breastfeeding at the time of the survey, the child’s age at that time was used to indicate the time when they had not experienced the ‘event’ or ceasing breastfeeding. Duration of EBF was derived from the age when other foods or liquids apart from breast milk were introduced. Questions related to infant feeding practices were based on the Australian National Infant Feeding Survey [23] and modified to include other fluid options commonly introduced by Chinese families as described. The suitability of these modifications was confirmed through face validity and test-retest reliability.

#### Exposure measures

The selection of exposure variables included maternal, psychosocial and cultural factors, demographic, child and birth factors, and maternal health factors that have been shown or hypothesised to be important based on current literature.

##### Feeding intentions and awareness of feeding recommendations

Participants were asked to recall how they intended to feed their baby from birth selecting from pre-specified options ranging from *breastfeeding only*, *combined breastfeeding with water or juice or formula*, to *formula feed only* or *other*. Participants also indicated their intended duration of any breastfeeding from given options of *at least 6, 12, 24 months* or *planned to breastfeed for as long as possible* or *didn’t think about how long*.

Awareness of the recommended infant feeding practices was assessed by two questions. The definition of EBF was provided and participants were asked what they thought the recommended duration of EBF was, and what the recommended age to introduce solids was. Participants could answer in weeks, months or *don’t know*.

##### Breastfeeding attitudes, control and perceptions of breastfeeding norms

Breastfeeding attitudes, control and perceptions of breastfeeding norms were measured using a modified version of the Breastfeeding Attrition Prediction Tool (BAPT), first developed and validated by Janke (24) for use in mainly white mothers. The original tool included 52 items on a 6-point Likert type scale and four subscales: Negative Breastfeeding Sentiment, Positive Breastfeeding Sentiment, Social and Professional Support, and Breastfeeding Control. A higher score indicates better attitude, greater perceived social pressure to breastfeed, and higher confidence to breastfeed.

Several items were modified to suit the Chinese Australian context. In the Social and Professional subscale, the questions asked the mother to rate how much she perceived certain social and professional contacts want her to breastfeed from ‘definitely not breastfeed’ to ‘definitely breastfeed’ or ‘not applicable’. The options were modified to suit the Chinese Australian context and included family members (baby’s father, my mother, my mother in law, my sister, other relatives), friends (my closest female friend), and health professionals (maternal and child health nurse, my doctor, my midwife). During analysis, the responses for this subscale was recoded to − 3 to 3 including zero to indicate non-applicability for ease of interpretation. The positive breastfeeding sentiment subscale was excluded as in the original study there was a general tendency for women to agree with the items resulting in decreased variability and poor predictive ability [24]. The modified BAPT tool consisted of 15 items for negative breastfeeding sentiment (disadvantages of breastfeeding and advantages of formula feeding), 9 items measuring subjective norm (mother’s perception on whether health professionals and their social contacts believed she should breastfeed or not), and 10 control items (perception of ease or difficulty associated with breast and formula feeding).

Confirmatory factor analysis was conducted on the modified BAPT tool to check if these constructs were adequately represented in Chinese Australian mothers. The results of the factor analysis suggested several modifications were necessary. An item (“I didn’t need help to breastfeed”) in the control scale was excluded due to poor factor loading (0.27). The subjective norm construct was a better fit as two factors, namely family and health professionals. Two items (“my sister” and “other relatives”) in the subjective norm family scale were excluded due to the high proportion of non-applicable responses.

The Cronbach’s alpha coefficient for the modified BAPT tool in this study with 289 participants were 0.93 for the attitude scale, 0.71 for the subjective norm family members, 0.92 for the subjective norm health professionals, and 0.91 for the control scale, indicating high internal consistency. This is comparable to the internal consistency of the original tool of 0.83, 0.85, and 0.81 respectively.

##### Cultural factors

Participants were asked if they followed the traditional Chinese postnatal confinement practice and whether they thought this traditional practice was important in helping a mother to breastfeed using a 5-point scale ranging from ‘extremely important’ to ‘not at all important’. Acculturation level was measured with a single question stating *what language do you prefer?* with five response options (Chinese only, mostly Chinese and some English, Chinese and English equally well, mostly English and some Chinese, English only) [25].

##### Valued sources of information

Participants were asked to rate the relative importance on a 5-point scale (extremely important to not at all important or not applicable if they did not receive any advice) of various sources of information in influencing their feeding decisions. Sources included the woman’s partner, her mother, mother in law, other relatives, friends, other mothers in the community, health professionals (maternal child health nurses, midwives, doctors, other hospital staff, lactation consultant, antenatal class), breastfeeding support hotlines, and the internet (website, blogs, and apps).

##### Demographic, maternal health and child factors

Basic maternal and child demographical details were collected including date of birth, child’s gender, maternal marital status (re-categorised to married and not married with the latter category consisting of de facto, divorced, separated, never married, and widowed), maternal country of birth, maternal employment status (full time or part time including self-employed, or not working or studying including those who were unemployed or caring for children full time), and indicators of socioeconomic status including maternal education levels, family annual income, and postcode (categorised according to socioeconomic indexes for areas). Additional details important to breastfeeding asked were infant’s age when mothers returned to work, the length of residence in Australia, if they co-resided with either maternal or paternal grandparents at any time during the child’s first year of life, and the number of children (re-categorised to primipara and multipara).

Information related to maternal health known to influence breastfeeding were also collected. These included maternal smoking status during pregnancy, presence of diabetes (pre-existing and gestational diabetes), pre-pregnancy weight and height (converted to body mass index and categorised into healthy, overweight, or obese according to World Health Organization’s cut-off values for Asian populations [26]), and self-report diagnosis of postnatal depression or anxiety (yes/no/unsure). Similarly, relevant obstetric factors such as child’s birth weight, and mode of delivery were collected.

#### Survey validity and reliability

Face validity was conducted for the whole survey with 10 people from the target group and several experts in the topic area. Minor suggestions were made related to the readability and flow of the survey, which was subsequently revised. Survey reliability was determined using the test-retest method where a subset of 50 participants were asked to complete the questionnaire again within 2 weeks of first completion [27]. The retest sample reflected participants who have stayed in Australia for longer (15 vs. 12 years) and whose language preference was bilingual or predominantly English. No other differences in demographic characteristics were found. Cohen’s Kappa or two way mixed effect model of intraclass correlation (ICC) [28] were used to quantify the reliability of the responses. Reliability coefficients for infant feeding practices (ICC 0.70–0.97; Kappa 0.70–1.00), psychosocial (ICC 0.51–0.82; Kappa 0.50–0.67) and cultural factors (Kappa 0.61–0.89) indicated moderate to high reliability. Responses to the importance of various sources of information for infant feeding had fair to moderate reliability (Kappa 0.35–0.58) with responses related to family members showing better reliabilities than other categories. The interpretation of the ICC and Kappa coefficient was based on cut offs suggested by Koo and Li [28] and Landis and Koch [29] respectively.

#### Statistical analysis

Descriptive analysis was performed for infant feeding practices in the first 6 months and determinants including demographic, psychosocial, cultural characteristics, maternal health, child and birth factors. The proportion of ‘any breastfeeding’ was determined using survival analysis. Multivariable logistic regression was used to examine the key factors associated with EBF at 4 months and the introduction of formula in the first month of life. Variables that were associated with the outcomes in the univariable analysis with *p* < 0.1 were included in the multivariable model to assess their independent effects [30]. All factors were checked for multicollinearity prior to multivariable modelling. The final model was checked using Hosmer and Lemeshow goodness of fit test. Data were analysed using Stata, version 15.0 software.

## Results

### Participant’ characteristics

A total of 334 mothers completed the survey, 45 were excluded as they did not meet the child age inclusion criteria, yielding final samples of 289. Participant characteristics (Table 1) reflected an older and socioeconomically advantaged cohort. The mean maternal age was 34 years with a child aged 2 years, and just under half (48%) were primiparas. The majority of participants were married, had tertiary education, were born in mainland China, were non-smokers and had a median length of stay in Australia of 10 years. Over half (60%) of the participants were employed and the mean child’s age when these mothers returned to work was 10 months. A majority (72%) had an elder (maternal or paternal parents) living together at some point in the first year postpartum.

**Table 1:** Participant characteristics (*N* = 289^a^)

Characteristics	No (%)
***Socio-demographic factors***	
**Maternal Age (years)** mean (SD^b^) range	*34 (4.0) 23–45*
**Socioeconomic Indexes for Areas**	
Greatest disadvantage (1st - 2nd quintile)	50 (19.5)
Moderate disadvantage (3rd - 4th quintile)	98 (38.1)
Least disadvantage (5th quintile)	109 (42.4)
**Marital Status**	
Married	241 (92.0)
Not married	21 (8.0)
**Education**	
< tertiary	40 (15.3)
Tertiary	222 (84.7)
**Employment**	
Full time	65 (24.8)
Part time	92 (35.1)
Not working or studying	105 (40.1)
**Child’s age (months) when mothers returned to work**	
Mean (SD) range	*10 .0 (5.2) 1–34*
**Family Income**^**c**^	
Low (<$50,000)	43 (16.4)
Middle ($50,000 - $79,999)	51 (19.5)
High (>$80,000)	117 (44.7)
Chose not to answer	51 (19.5)
**Maternal Birth Region**	
Mainland China	123 (46.8)
Hong Kong	53 (20.2)
Taiwan	63 (24.0)
Australia	12 (4.6)
Other (Malaysia, Indonesia, Vietnam)	12 (4.6)
**Length of residence in Australia**	
Median (IQR) years	*10 (6–15)*
**Co-resided with grandparents during the first 12 months of life**	
Yes	189 (71.6)
No	75 (28.4)
***Maternal and obstetric factors***	
**Parity**	
Primipara	126 (48.3)
Multipara	135 (51.7)
**Smoking during pregnancy**	
Yes	7 (2.7)
No	255 (97.3)
**Pre pregnancy body mass index (kg/m**^**2**^**)**	
Underweight (< 18.5)	39 (13.5)
Healthy (18.5–23)	167 (57.8)
Overweight/Obese (≥24)	83 (28.7)
**Diabetes status**	
Pre-existing diabetes	7 (2.7)
Gestational diabetes	63 (24.1)
No	192 (73.2)
**Post-natal Depression**	
Yes	26 (9.9)
No	186 (71.0)
Unsure	50 (19.1)
**Mode of birth**	
Vaginal	214 (74.1)
Caesarean section	75 (25.9)
***Child factors***	
**Child’s age (years)** mean (SD) range	*2.0 (0.7) 1.0–3.9*
**Child’s gender**	
Male	136 (47.1)
Female	153 (52.9)
**Child’s birth weight (kg) mean (SD) range**	*3.3 (0.5) 2.0–5.0*

^a^ total sample varied between 262 and 289 due to missing data

^b^*Abbreviations*: *SD* Standard deviation, *IQR* Interquartile range

^c^ All figures are in Australian dollars

### Psychological and cultural factors

Table 2 describes breastfeeding related psychological and cultural factors. More than half (61%) of mothers intended to exclusively breastfeed at birth, while another 39% intended to mix feed with formula. Half of the mothers intended on maintaining breastfeeding to 6–12 months with another 30% wanting to breastfeed for as long as possible. Less than half (44%) of mothers correctly stated that the recommended EBF duration was 6 months. Similarly, 53% of mothers thought that the appropriate age to introduce solid foods was 6 months. The mean scores of the BAPT scales showed a low-moderate level of negative breastfeeding sentiment, a moderate-high level of breastfeeding control, a very high perceived breastfeeding norm from health professionals and a high perceived breastfeeding norm among family members. The majority of the participants were less-moderately acculturated with only 10% of mothers preferring mostly English. More than 3 out of 4 (77%) participants followed the Chinese traditional postnatal care and a further 5% of the participants were not able to follow it but would have liked to. Following Chinese traditional postnatal care was thought to be highly important to breastfeeding by 59% of the participants.

**Table 2:** Description of breastfeeding related psychological and cultural predictors

Factors	***N*** (%) or mean (SD)
**Psychological factors**	
**Feeding intention at birth**	
Breastfeed only	176 (60.9)
Mix feed with formula	112 (38.8)
Formula feed only	1 (0.4)
**Intention of ‘any breastfeeding’ duration**	
At least 6 months	101 (30.6)
At least 12 months	68 (20.6)
At least 24 months	16 (4.8)
As long as possible	100 (30.3)
Didn’t think how long	45 (13.6)
**Awareness of exclusive breastfeeding recommendation**	
6 months	127 (44.3)
Others (don’t know and ≠6 months)	160 (55.8)
**Awareness of recommended age to introduce solid foods**	
6 months	154 (53.3)
≠ 6 months	135 (46.7)
**Negative breastfeeding sentiment score**	226.7 (100.2) possible scores 15–540
**Breastfeeding control score**	40.5 (10.4) possible scores 9–54
**Health professional subjective norm score**	25.7 (19.2) possible scores −27 to 27
**Family subjective norm score**	20.2 (21.3) possible scores −36 to 36
***Cultural Factors***	
**Preferred Language**	
Predominantly Chinese	98 (37.4)
Bilingual	137 (52.3)
Predominantly English	27 (10.3)
**Followed Chinese traditional postnatal care**	
Yes	219 (76.6)
No and wouldn’t like to	53 (18.5)
No but would like to	14 (4.9)
**Importance of traditional postnatal care to breastfeeding**	
Extremely important	80 (24.5)
Very important	112 (34.3)
Moderately important	65 (19.9)
Slightly important	39 (11.9)
Not at all important	31 (9.5)

### Infant feeding practices

**Fig. 1:**
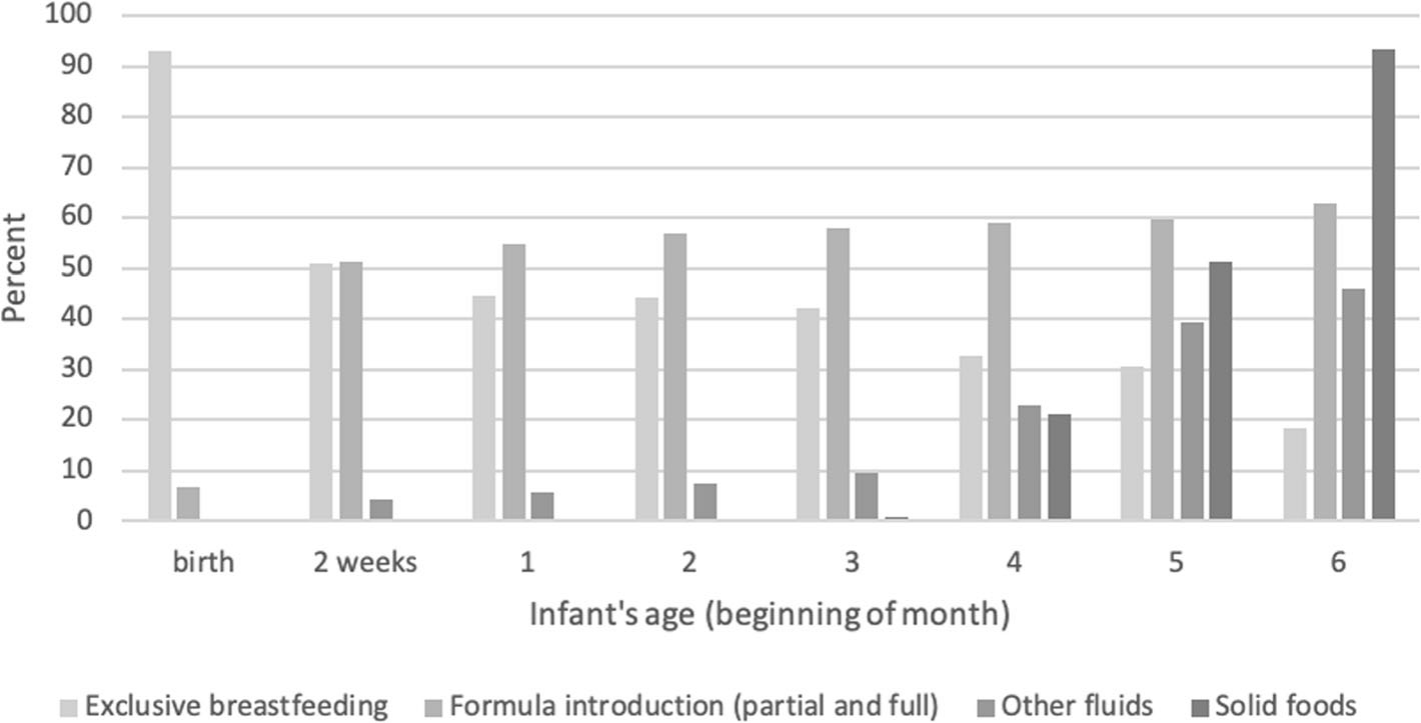
Percentage of infants exclusively breastfed, introduced to formula, ‘other fluids’, and solid foods in the first 6 months

Key infant feeding practices in the first 6 months of life are shown in Fig. 1. At birth, 93% of mothers initiated EBF. By 1 month of age, EBF rates had dropped to 44% and concurrently formula use increased to 55%. For mothers who ceased EBF within the first month, the median age of introducing formula was 3 days. At 4 months of age, 33% of mothers continued to breastfeed their infants exclusively. Of the mothers who initiated EBF at birth, the median duration of EBF was 20 (IQR 2–151) days. The introduction of ‘other fluids’ was minimal (6%) in the first month of life increasing to 10% at 3 months. From 4 months of age, solid foods and ‘other fluids’ were increasingly introduced such that at 6 months of age 18% of mothers were exclusively breastfeeding.

**Fig. 2:**
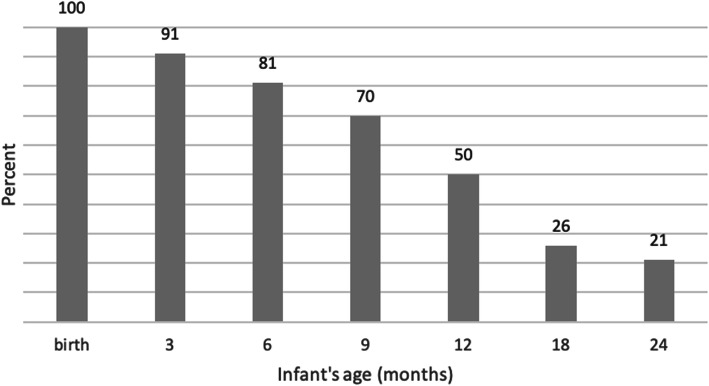
Proportion of infants receiving any breast milk at each month of age

All infants in this study were breastfed at birth (either exclusively, or in combination with formula). The rate of ‘any breastfeeding’ was 81% at 6 months of age (Fig. 2). This reflects a high prevalence of mixed feeding, predominantly with formula in the first 3 months then at 4–6 months with formula, solid foods and ‘other fluids’. At 12 months of age around half of the infants continued to receive some breast milk while at least one in five children continued to receive breast milk at 2 years of age.

#### Factors associated with exclusive breastfeeding at 4 months

This study aimed to examine the factors associated with EBF at 6 months, however, due to small numbers (53 or 18%), a 4-month cut-off was used for this analysis. In multivariable analysis, the odds of EBF at 4 months were increased with higher breastfeeding control (aOR 1.07; 95% CI 1.03, 1.11) and longer length of residency in Australia (aOR 1.05; 95% CI 1.00, 1.10). However the odds were reduced with intention to mix feed with formula from birth (aOR 0.24; 95% CI 0.12, 0.50), and a lack of awareness of the recommended EBF duration (aOR 0.40; 95% CI 0.20, 0.80) and the recommended age to introduce solids (aOR 0.48; 95% CI 0.25, 0.93). See Table 3 for complete model results.

**Table 3:** Predictors of exclusive breastfeeding at 4 months^a^

Factors	Univariable analysis^**b**^		Multivariable analysis	
	OR	(95% CI)	aOR	(95%CI)
**Psychological factors**				
**Feeding intention at birth**				
EBF only	1		1	
Mix feed with formula	0.21***	0.12, 0.38	0.24***	0.12, 0.50
**Awareness of EBF recommendation**				
6 months	1		1	
Others (don’t know and ≠6 months)	0.34**	0.20, 0.56	0.40*	0.20, 0.80
**Awareness of recommended age to introduce solid foods**				
6 months	1		1	
≠ 6 months	0.58*	0.35, 0.95	0.48*	0.25, 0.93
**BAPT**				
Negative breastfeeding sentiment score	0.996**	0.994, 0.999	1.000	0.996, 1.004
Breastfeeding control score	1.07***	1.04, 1.10	1.07***	1.03, 1.11
Family subjective norm score	1.02*	1.00, 1.03	1.01	0.99, 1.03
**Cultural factors**				
**Language preference**				
Predominantly Chinese	1		1	
Bilingual	1.89*	1.06, 3.35	1.44	0.67, 3.09
Predominantly English	2.47*	1.02, 5.99	1.25	0.34, 4.60
**Socio-demographic factors**				
**Employment**				
Full time	1		1	
Part time	1.97	0.98, 3.98	1.61	0.66, 3.91
Not working or studying	1.60	0.80, 3.20	1.71	0.68, 4.32
**Family Income**^**c**^				
High ($80,000 and over)	1		1	
Middle ($50,000 - $79,999)	0.63	0.31, 1.29	0.82	0.33, 2.02
Low (<$50,000)	0.44*	0.19, 1.00	0.61	0.23, 1.67
Wish not to answer	1.16	0.59, 2.27	1.25	0.52, 3.05
**Length of residence in Australia (years)**	1.05*	1.02, 1.08	1.05*	1.00, 1.10
**Maternal health and obstetric factors**				
**Mode of birth**				
Vaginal	1		1	
Caesarean	0.53*	0.29, 0.96	0.83	0.38, 1.80
**Pre-pregnancy body mass index**	0.93	0.85, 1.01	0.99	0.88, 1.11

*Abbreviations*: *CI* Confidence interval, *OR* Odds ratio, *aOR* Adjusted odds ratio, *EBF* Exclusive breastfeeding, *BAPT* Breastfeeding attrition prediction tool

* *p* < 0.05

**p < 0.01

***p < 0.001

^a^ Sample size varied were between 262 and 289 due to missing data

^b^ Variables with *p* value greater than 0.1 in univariable analysis were excluded in the multivariable analyses. These variables were: intention of ‘any breastfeeding’ duration, maternal age, SEIFA, parity, diabetes status, postnatal depression status, child’s gender and birthweight, health professional subjective norm score, traditional postnatal care practise, importance of traditional postnatal care, co-residence with grandparents

^c^ All figures are in Australian dollars

#### Factors associated with the introduction of formula by 1 month of age

The odds of introducing formula by 1 month of age were increased by maternal intention to mix feed with formula from birth (aOR 4.21; 95% CI 2.22, 7.97) and a low family income (aOR 3.18; 05% CI 1.24, 8.15). On the other hand, mothers who had higher breastfeeding control (aOR 0.94; 95% CI 0.91, 0.97), and who were not working (aOR 0.37; 95% CI 0.15, 0.89) were less likely to introduce formula by 1 month of age. See Table 4 for complete model results.

**Table 4:** Predictors of the introduction of formula by 1 month of age^a^

Factors	Univariable analysis^b^		Multivariable analysis	
	OR	(95% CI)	aOR	(95%CI)
**Psychological factors**				
**Feeding intention at birth**				
EBF only	1		1	
Mix feed with formula	4.85***	2.89, 8.17	4.21***	2.22, 7.97
**Awareness of EBF recommendation**				
6 months	1		1	
Others (don’t know and ≠6 months)	1.66*	1.04, 2.67	1.38	0.74, 2.57
**BAPT**				
Negative breastfeeding sentiment score	1.00**	1.00, 1.01	1.001	0.998, 1.004
Breastfeeding control score	0.94***	0.91, 0.96	0.94**	0.91, 0.97
Family subjective norm score	0.98*	0.97, 1.00	0.99	0.98, 1.01
Health professionals subjective norm score	0.99	0.98, 1.00	0.99	0.97, 1.01
**Cultural factors**				
**Language preference**				
Predominantly Chinese	1		1	
Bilingual	0.77	0.45, 1.30	0.90	0.44, 1.86
Predominantly English	0.44	0.18, 1.04	0.46	0.13, 1.63
**Socio-demographic factors**				
**Employment**				
Full time	1		1	
Part time	0.48*	0.25, 0.94	0.58	0.25, 1.29
Not working or studying	0.45*	0.24, 0.87	0.37**	0.15, 0.89
**Family Income**^**c**^				
High ($80,000 and over)	1		1	
Middle ($50,000 - $79,999)	1.71	0.87, 3.36	1.94	0.83, 4.54
Low (<$50,000)	2.35*	1.11, 4.94	3.18**	1.24, 8.15
Chose not to answer	1.06	0.55, 2.04	1.48	0.64, 3.43
**Length of residence in Australia**	0.97	0.94, 1.00	0.99	0.95, 1.04
**Maternal health and obstetrics factors**				
**Mode of birth**				
Vaginal	1		1	
Caesarean	1.78*	1.03-, .08	1.50	0.74, 3.05
**Pre-pregnancy body mass index**	1.11*	1.02, 1.21	1.09	0.98, 1.20

*Abbreviations*: *CI* Confidence interval, *OR* Odds ratio, *aOR* Adjusted odds ratio, *EBF* Exclusive breastfeeding

* *p* < 0.05

**p < 0.01

***p < 0.001

^a^ Sample size varied were between 262 and 289 due to missing data

^b^ Variables with *p* value greater than 0.1 in univariable analysis were excluded in the multivariable analyses. These variable were: intention of ‘any breastfeeding’ duration, awareness of timing to introduce solid foods, maternal age, marital status, SEIFA, education level, parity, diabetes status, postnatal depression, child’s gender and birthweight, practised traditional postnatal care, importance of traditional postnatal care, co-residence with grandparents

^c^ All figures are in Australian dollars

#### Valued sources of information

Table 5 details the importance of various sources of information including family, health professionals, community, and online breastfeeding information in influencing mothers’ infant feeding decisions. The top five information sources that were rated as extremely or very important were all types of health professionals, especially maternal and child health nurses or midwives, lactation consultants and doctors, followed by the women’s partners. Moderately important sources of information were women’s own mothers, friends and other mothers in the community, websites or blogs and apps and women’s partners. The least important sources of information were relatives, mother in law, friends, and other mothers. The sources of information with the highest percentage of women who did not receive infant feeding advice from were breastfeeding helpline (28%), relatives (26%), apps (21%) and antenatal class (20%).

**Table 5:** The importance of various sources of information in influencing infant feeding decisions of Chinese Australian mothers

	Extremely Important	Very Important	Somewhat important	Slightly important	Not important	Not applicable
Family and relatives						
Partner	**20.13**	24.42	**29.7**	13.2	3.96	8.58
Mother	12.54	24.42	**35.31**	12.21	**5.94**	9.57
Mother in law	3.63	14.52	27.72	**23.76**	**12.87**	**17.49**
Relatives	1.65	6.27	15.84	**28.38**	**22.11**	**25.74**
Community						
Friends	2.64	9.24	**33.99**	**24.75**	**11.88**	**17.49**
Other mothers	7.59	22.77	**35.97**	**13.86**	**8.25**	11.55
Health professionals						
MCH nurse or midwives	**23.43**	**40.59**	24.42	5.61	2.97	2.97
Doctor	**21.45**	**35.64**	26.73	4.62	3.96	7.59
Antenatal class	**18.48**	**27.39**	22.77	6.6	4.62	**20.13**
Hospital staffs	**18.48**	**29.37**	27.72	6.93	3.96	13.53
Lactation consultant	**28.71**	**30.03**	15.18	4.95	3.63	**17.49**
Other						
Breastfeeding helpline	17.16	23.43	16.17	10.89	4.29	**28.05**
Websites/blogs	14.52	25.74	**33.99**	10.23	4.29	11.22
Apps	10.56	18.15	**29.70**	**15.51**	4.95	**21.12**

Bold typeface indicates the five highest percentages in each categories of importance

*MCH* Maternal and child health

## Discussion

This is the first study to examine the psychosocial and cultural factors of early introduction of formula and duration of EBF in Chinese Australian mothers. This study found that 55% of mothers had introduced formula in the first month postpartum yet continuation of ‘any breastfeeding’ remained high across the first 12 months of life. This indicates that formula is used to supplement rather than to replace breastfeeding. The odds of early introduction of formula and continuation of EBF were significantly associated with both breastfeeding intention at birth and breastfeeding control. Continuation of EBF to 4 months was also predicted by an awareness of infant feeding recommendations and longer residency in Australia. Low income families and working mothers are at higher risk of early introduction of formula. Chinese Australian mothers valued the information mostly from health professionals followed by immediate family (partner and maternal mother), social networks and the internet.

Compared to the Australian national breastfeeding rates in 2010, a slightly lower proportion of Chinese Australian mothers exclusively breastfed to 4 months (33% vs. 39%) and twice as many had introduced formula by 1 month (55% vs. 27%) [7]. However, a higher proportion of Chinese Australian women consistently offered any breast milk at each month in the first 2 years than the general Australian population (for example: 91% vs. 73% at 3 months, 81% vs. 63% at 6 months, 50% vs. 42% at 12 months, 21% vs. 7% at 24 months). Although not directly comparable due to differences in the definition of ‘Chinese’ ethnicity and representativeness, our study confirmed a previous study which analysed the national infant feeding survey data, that on average Chinese born women introduced formula at a younger age than Australian born women, but that they tended to breastfeed for longer [8].

The high rate of mixed feeding in Chinese Australian mothers is consistent with other studies of Chinese women in Australia, Canada, the United Kingdom and China [8, 31–33]. This study further adds that the most vulnerable period for formula supplementation among Chinese Australian mothers is within the first three days postpartum. This is the time when the onset of copious milk secretion, known as lactogenesis II, is happening [34]. Chinese Australian mothers may perceive that they have insufficient milk supply and resort to supplementing with formula, when in fact lactogenesis II is in progress. While evidence indicates that mothers with delayed lactogenesis II (> 72 h postpartum) are more likely to cease EBF prematurely [35], results from large cohort studies in China show that only 9–14% of mothers experience delayed lactogenesis II [36, 37]. Mothers may be unaware of the timing and process of the onset of lactogenesis II, resulting in heightened anxiety about milk supply and subsequent formula supplementation. Support needs to be given to prepare and support mothers regarding normal physiological process of lactation and encourage them to feed frequently to increase milk supply.

While the introduction of formula was commonplace, the high rates of ‘any breastfeeding’ throughout the first year of life suggest that Chinese Australian mothers are committed to breastfeeding. This is important to acknowledge and highlights opportunities to enhance EBF and breastfeeding maintenance across the first year of life. Exclusive breastfeeding appeared to be significantly influenced by maternal feeding intention at birth, which is consistent with the literature [11, 38, 39]. It is worth noting that in this study more than one in three mothers reported intention to mix breast and formula feed from birth. According to the theory of planned behaviour, the intention to perform a behaviour is predicted from attitudes towards the behaviour, perceived control of internal and external barriers to performing the behaviour and the perceived social pressure to perform (or not) the behaviour [40]. These constructs have been shown to be important in explaining breastfeeding intentions and practices in the Chinese population [41–43]. Given the high prevalence of formula consumption in China [44], there may be a social norm of supplementing with formula promoted through the media or social networks. This may mean that many Chinese Australian mothers do not have a strong role model and advocate for breastfeeding and hence are more accepting of mix feeding. Creating opportunities to learn from breastfeeding role models prior to giving birth may be helpful in fostering a stronger intention and confidence to breastfeed exclusively.

An awareness of the infant feeding recommendations, including the duration of EBF and the age of introduction of solid foods, was important in sustaining EBF for Chinese Australian mothers. Previous studies have reported an association between awareness of feeding guidelines and intention to breastfeed exclusively [45, 46]. It has been reported that 80% of pregnant women in China were not aware of the EBF duration guideline [46] and that early introduction of solid foods prior to 4 months was common [47]. Although the proportion of Chinese mothers who were not aware of the feeding recommendations in this study were lower in Australia compared to in China, it is still quite high. The practice of introducing solid foods early appears to be rooted in tradition with historical writing recording advice to give rice water or other foods to infants from 1 month of age [48]. These findings suggest that there is a need to better understand how to improve awareness of the recommendations around EBF and timing of solid foods prior to birth as well as support to implement them after birth.

Breastfeeding control, a measure of mother’s self-efficacy for breastfeeding, directly predicted breastfeeding exclusivity from birth to 4 months. The relationship between breastfeeding self-efficacy and EBF among Chinese mothers has been frequently reported [49–52]. Breastfeeding self-efficacy is facilitated by greater social support to breastfeed, the ability to follow traditional postnatal care practices in those who valued the practice, access to trusted or preferred health practitioners to help with breastfeeding problems, and an understanding of healthy infant growth [53–55]. On the other hand, conflicting breastfeeding beliefs and practices between women’s original culture and the recommendations received in Australia have been reported to undermine confidence to breastfeed exclusively [56]. Studies have reported that Chinese mothers often receive pressure to supplement with formula from family members (women’s partners, mother or mother-in-law) to ensure that the infant is sufficiently nourished [54, 57].

In this study, living together with the child grandparent(s) in the early postnatal period was common and mothers who co-resided with the child’s grandparent(s) could have experienced more pressure to supplement with formula. A previous study with Chinese women in Hong Kong found that living with a parent in law was associated with ceasing breastfeeding before 1 month [58]. However, the current study did not find evidence of any relationships between co-residence with grandparent(s) and EBF or formula introduction. Unlike the study in Hong Kong, the current study did not differentiate whether women were living with their parents or parents in law, which may partly explain the null findings. In traditional Chinese culture, a woman’s mother in law typically carries a lot of power in the family and expects the new mother to respect and obey her wishes, including on how to feed the infant [59]. However, the power dynamic was much different if it was their mother, and they would have more room to negotiate on how to feed the baby [60].

In addition, Australia promotes a culture of greater autonomy to women compared to Chinese culture which values women who sacrifice their individual wishes to achieve family harmony. It is possible that with greater acculturation in Australia, mothers were more assertive to their female elders to maintain EBF and able to resist the pressure to supplement with formula. A recent study with Chinese immigrant mothers in the United States found that while personal beliefs were most predictive of feeding intentions in the overall sample, social norms (how others want her to feed) most strongly predicted feeding intentions among the least acculturated Chinese immigrant mothers [61]. Our study also found a significant association between longer length of residency in Australia and EBF to 4 months. Although greater acculturation may result in improved feeding practices among Chinese immigrant mothers, they may also experience greater family conflicts due to unresolved differences with others who hold on to a different view [62]. There is a need to explore how Chinese Australian women should be supported to increase their breastfeeding control while maintaining family harmony.

An unexpected finding from this study was the significant association between employment status at 1–4 years postpartum and early introduction of formula by 4 weeks of age. Other studies in China and Hong Kong have consistently linked employment with short breastfeeding duration and exclusivity [37, 39]. The association could be explained by short maternity leave where the majority of women in China return to work when their infants were aged around 2 to 3 months, consequently infants were fed with formula. The mothers in this study returned to work on average when their infant was 10 months old, hence unlikely to explain formula introduction at 1 month of age. However, Chinese mothers may be concerned that an exclusively breastfed baby is hard to wean, which will limit their flexibility to return to work later on. A previous study has reported that there was a common perception among Chinese mothers that they need to ‘train’ their baby to accept both breast milk and formula from an early age to make the weaning process easier when mothers want to return to work [53].

Given the high regard to advice provided by health professionals, women’s partner and their mother, health services’ breastfeeding support should include these family members to facilitate better communications regarding infant feeding. In particular, education could be provided to address their ambivalence regarding supply issues and appropriate ways to resolve feeding problems [63, 64]. Furthermore, the internet was found to be a prominent source of information among Chinese Australian mothers, which implies that there is potential to use this avenue to promote evidence based infant feeding information and support.

### Strengths and limitations

This study investigated a broad range of psychosocial and cultural factors in relation to formula and breastfeeding practices among a relatively large sample of Chinese Australian mothers. There are several limitations to note. The retrospective data collection cannot confirm temporal relationships between breastfeeding attitude, perception of social breastfeeding norms and control measures and breastfeeding intention. Hence, the possibility of reverse causation could not be ruled out. The estimation of EBF duration in this study was derived as the age in which other foods or fluids apart from breast milk were introduced. Given that maternal recall of the age of introduction of foods and fluids other than breast milk was known to be less reliable [22], the estimation of EBF duration could be less accurate. The test-retest study showed moderate reliability for the measure of the age of introduction of ‘other fluids’ but excellent reliability for the age of formula introduction. Since the vast majority of mothers in this study ceased EBF due to the introduction of formula rather than ‘other fluids’, the estimation of EBF duration should remain robust. Despite our intention, this study could not examine the factors associated with EBF at 6 months as relevant to the infant feeding guidelines due to insufficient numbers. Another limitation related to the potential underestimation of any breastfeeding rates at 18 and 24 months, given that 55 children aged under 2 years were being breastfed at survey completion. Lastly, while effort was given to recruiting a wide range of participants, there were difficulties in reaching Chinese Australian mothers with lower socioeconomic status. Hence, care should be taken when generalising the findings to the wider Chinese Australian population with different demographic characteristics.

## Conclusion

There was a high prevalence of EBF attrition among Chinese Australian mothers from early days postnatal but this was coupled with sustained duration of ‘any breastfeeding’ in the first year of life. This study expands our understanding of how Chinese Australian mothers can be supported to establish and maintain EBF. Maternal breastfeeding intention, self-efficacy and awareness of infant feeding recommendations were found to be important factors associated with EBF from birth to 4 months. These modifiable factors provide focus for considerations in which health service providers might seek to support EBF in this community.

## Data Availability

The datasets generated and/or analysed during the current study are not publicly available due to conditions of ethics approval but are available from the corresponding author on reasonable request.

## Abbreviations

EBF: Exclusive breastfeeding
BMI: Body mass index
SEIFA: Socio-Economic Indexes for Areas
